# Bent Dinuclear Platinum(II) Halo-Bridged Carbonyl Complexes

**DOI:** 10.3390/molecules16076082

**Published:** 2011-07-20

**Authors:** Daniela Belli Dell'Amico, Luca Labella, Fabio Marchetti

**Affiliations:** Dipartimento di Chimica e Chimica Industriale, Università di Pisa, via Risorgimento 35, I-56126 Pisa, Italy

**Keywords:** platinum, dinuclear complexes, halo-bridged complexes

## Abstract

Crystals of *trans*-Pt_2_(*μ*-X)_2_X_2_(CO)_2_ (X = Br, I) have been grown and their molecular and crystalline structures have been solved by X-ray diffraction methods. In both cases the dinuclear molecules are bent, with a bending angle of 164.6° and 156.5° for the bromide and the iodide, respectively. While the structure of the bromo-derivative is reported here for the first time, a modification of *trans*-Pt_2_(*μ*-I)_2_I_2_(CO)_2_ with planar centrosymmetric molecules is known. This appears to be a rare case of a platinum(II) halo-bridged derivative structurally characterized in both bent and planar forms.

## 1. Introduction

**Figure 1:**
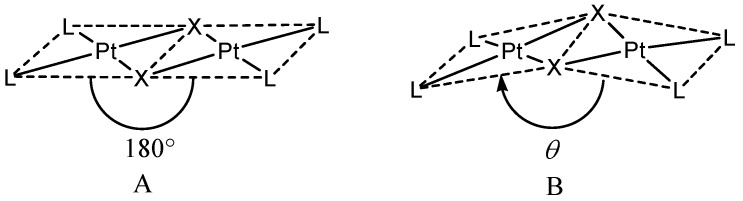
Planar (**A**) or bent (**B**) edge-sharing binuclear complexes of platinum(II).

Halo-bridged dinuclear derivatives of palladium(II) and platinum(II) [1,2] usually show square coordination geometries around the metal centres, with a *θ* angle between the two coordination planes (see Figure 1A) equal or close to 180°, corresponding to a planar or quasi-planar molecular arrangement.

Nevertheless, some exceptions are known [3,4,5] and bent molecules showing *θ* minor than 160° have been structurally characterized. Among the bent halo-bridged complexes of *d*^8^ transition metal ions the rhodium(I) carbonyl Rh_2_(*µ*-Cl)_2_(CO)_4_ is well-known (*θ* = 124°) [6,7]; in this complex the presence of a metal−metal bond has been suggested, in view of the rather short intra-molecular Rh−Rh distance (3.12 Å).

A theoretical investigation concerning the geometry of edge-sharing binuclear square complexes of *d*^8^ transition metal ions, enriched with a comparative analysis of the available experimental structural data, has been reported some years ago [1]. The authors concluded that the tendency to form bent structures increases this way: (a) concerning the metal centre, when descending along a group of the periodic table and from right to left along a period, (b) in the presence of two good *σ*-donor (and preferably good *π*-acid) terminal ligands per metal atom (in absence of steric hindrance) and (c) as the electronegativity of the bridging atoms decreases.

A search on deposited structures in the Cambridge Crystallographic Data Base [8] with formulae [L_2_Pt(*μ*-X)_2_PtL_2_] limited to the halo-bridged items, gave 74 results: 46 chloro-, 20 iodo- and eight bromo-complexes. In 64 of them the coordination squares of the two platinum centres in the molecule are coplanar (or quasi-coplanar), but in eight of them the molecules are bent, showing a *θ* angle significantly less than 180° [3,4,5,9,10,11,12,13,14]. Among the halocarbonyl complexes of platinum(II) [15] two dinuclear halo-bridged derivatives, namely *trans*-Pt_2_(*μ*-Cl)_2_Cl_2_(CO)_2_ [16,17] and *trans*-Pt_2_(*μ*-I)_2_I_2_(CO)_2_ [18], have been structurally characterized and both complexes appear to be essentially planar.

Here we present the crystal and molecular structure of a second structural isomer of *trans*-Pt_2_(*μ*-I)_2_I_2_(CO)_2_, which shows a bent geometry, and the first crystallographic characterization of the isotypic *trans*-Pt_2_(*μ*-Br)_2_Br_2_(CO)_2_.

## 2. Results and Discussion

In previous studies [18] it has been observed that PtI_2_(CO)_2_ (predominantly the *trans* isomer), easily obtained by reacting PtI_2_ with CO at atmospheric pressure Equation (1), partially loses CO under vacuum Equation (2) affording the dinuclear *trans*-Pt_2_(*μ*-I)_2_I_2_(CO)_2_.

PtI_2_ + 2 CO → *trans*-PtI_2_(CO)_2_ (1)

2 *trans*-PtI_2_(CO)_2_ ⇌ *trans*-Pt_2_(*μ*-I)_2_I_2_(CO)_2_ + 2 CO (2)

The product, recrystallized from a toluene solution at room temperature under dinitrogen, and studied by X-ray single crystal diffraction, appeared to be planar and centrosymmetric [18].

**Figure 2:**
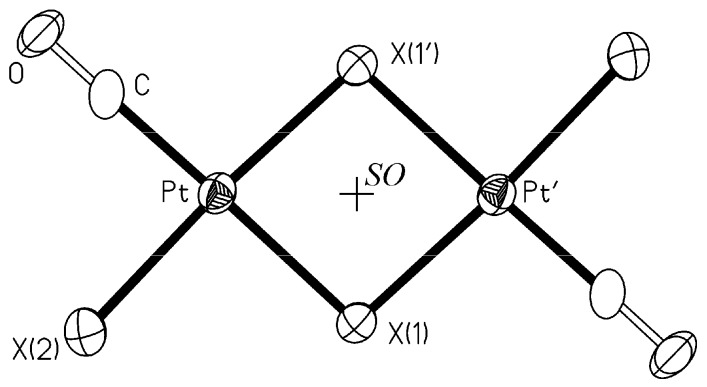
The molecular structure of *trans*-Pt_2_(*μ*-X)_2_X_2_(CO)_2_, X = Cl, Br, I. Thermal ellipsoids are at 30% probability. *SO* is the symmetry operator relating the two halves of the molecule: 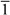 for X = Cl and I(*α*) and 2 for X = Br and I(*β*).

In the course of this work single crystals of the same product were obtained by recrystallization from toluene under CO atmosphere at −30 °C. Both *trans*-PtI_2_(CO)_2_ and *trans*-Pt_2_(*µ*-I)_2_I_2_(CO)_2_ were present in solution, according to equilibrium 2, the less soluble dinuclear complex precipitating out first. An X-ray diffraction experiment showed that we were dealing with a polymorph of *trans*-Pt_2_(*μ*‑I)_2_I_2_(CO)_2 _with a bent geometry (*θ* = 154.5°, see Figure 1B), named *β*-polymorph later on in the text. A view of the molecular structure is shown in Figure 2. Selected bond lengths (Å) and angles (°) are reported in Table 1.

The analogous bromo-derivative, *trans*-Pt_2_(*μ*-Br)_2_Br_2_(CO)_2_, has been prepared by refluxing solutions of *cis*-PtBr_2_(CO)_2_ in 1,2-dichloroethane under a gentle N_2_ flow to favour CO removal (see Equation (3).

2 *cis*-PtBr_2_(CO)_2_ ⇌ *trans*-Pt_2_(*μ*-Br)_2_Br_2_(CO)_2_ + 2 CO (3)

Crystals have been grown by slow evaporation of a solution of the derivative in CH_2_Cl_2_ at room temperature. No previous structural characterization of the product has been reported. An X-ray diffraction experiment has shown that the complex is bent (*θ* = 164.6°). The molecular structure is analogous to that observed in *β*-*trans*-Pt_2_(*μ*-I)_2_I_2_(CO)_2_, shown in Figure 2. Selected bond lengths (Å) and angles (°) are reported in Table 1.

Both *trans*-Pt_2_(*μ*-Br)_2_Br_2_(CO)_2_ and *β*-*trans*-Pt_2_(*μ*-I)_2_I_2_(CO)_2_, reported in this work, along with the already studied *trans*-Pt_2_(*μ*-Cl)_2_Cl_2_(CO)_2_ [16,17] and *α*-*trans*-Pt_2_(*μ*-I)_2_I_2_(CO)_2_ [18] show the expected molecular structure consisting in two edge sharing squares. All the four structures, as they appear in a projection on their mean plane, may be represented by the same sketch shown in Figure 2, the main difference being the symmetry operator (*SO*) relating the two squares.

In both *trans*-Pt_2_(*μ*-Cl)_2_Cl_2_(CO)_2_ and *α*-*trans*-Pt_2_(*μ*-I)_2_I_2_(CO)_2_ the symmetry operator is the inversion center 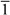, which imposes two coplanar squares, while in the isotypic bent molecules *trans*-Pt_2_(*μ*-Br)_2_Br_2_(CO)_2_ and *β*-*trans*-Pt_2_(*μ*-I)_2_I_2_(CO)_2 _the operator is the twofold axis.

The geometries of the four molecules are compared in Table 1, where the corresponding lengths and angles are listed in the same rows. All molecules show longer Pt−X bond distances for the bridging- with respect to the corresponding terminal halides, the difference decreasing from chloro- (0.215 Å) to iodo-complexes (0.030 and 0.017 Å for *α* and *β*, respectively). While in *trans*-Pt_2_(*μ*-Cl)_2_Cl_2_(CO)_2_ the Pt−Cl(1) distance (trans to CO) is significantly shorter than Pt−Cl(1*'*) (trans to terminal Cl), hardly significant differences (limit of 3*σ* ) are observed between the Pt−X bond distances for the bridging halides in the bromo- and iodo-complexes. A feature shared by the four molecules is the value of the bond angle X(1)−Pt−X(1*'*), which is constantly about 85°, regardless of the identity of X and the planar or bent shape of the molecule. Another common feature is the planarity of the square coordination around platinum, the maximum deviation being about 0.02 Å observed in *β*-*trans*-Pt_2_(*μ*-I)_2_I_2_(CO)_2_. The intramolecular Pt···Pt distances are in the range 3.4–3.8 Å, markedly longer than those expected in the presence of metal−metal bond (2.774 Å in the metal).

What distinguishes the two compounds described in this work from the other two already known [16,18], is the value of the *θ* angle (see Figure 1 and Table 1). 

**Table 1:** Selected bond lengths (Å) and angles (°) in the molecular structure of *trans*-Pt_2_(*μ*-X)_2_X_2_(CO)_2_.

	Cl^a^	Br^b^	I(*β*)^b^	I(*α*)^c^
Pt−X(1)	2.284	2.464(2)	2.608(3)	2.622(3)
Pt−X(1*'*)^ d^	2.392	2.457(2)	2.620(4)	2.630(2)
Pt−X(2)	2.123	2.411(3)	2.597(4)	2.596(3)
Pt−C	1.85	1.88(2)	1.84(4)	1.88(3)
C−O	1.07	1.12(2)	1.14(4)	1.06(4)
Pt⋯Pt*'*	3.441	3.579(1)	3.760(3)	3.846(2)
Pt⋯Pt*''*^,e^	3.525	3.741(2)	4.070(3)	--
X(1)−Pt−X(2)	93.96	90.34(8)	90.39(11)	90.68(10)
X(1)−Pt−X(1*'*)	85.29	85.65(8)	85.29(10)	85.83(10)
X(2)−Pt−C	89.41	88.6(7)	89.4(15)	87.0(8)
X(1*'*)−Pt−C	91.34	95.4(8)	94.9(16)	96.50(8)
Pt−X(1)−Pt*'*	94.71	93.31(7)	91.97(9)	94.17(10)
*θ*	180	164.6	156.4	180

^a^ ref. [16]; standard deviations are not reported as the structure of the chloride has been refined in an incorrect space group [17]. ^b^ this work; ^c^ ref. [18]; ^d^ Symmetry transformation used to generate equivalent atoms: *'* =1−*x*, −*y*, −*z* for X = Cl, −*x*, 1−*y*, *z* for X = Br and I(*β*) and −*x*, −*y*, −*z* for X = I(*α*),*''* = *x*, *y*, 1+*z* for X = Cl and −1/2+ *y*, 1/2+*x*, 1/2+*z* for X = Br and I(*β*), respectively; ^e^Pt⋯Pt distances among the nearest neighbours in the stacks.

In their survey on the *d*^8^-bent dimers with a core [M(μ-X)_2_M], Aullón *et al.* [1] attribute the driving force for bending to interactions between *d_z_*_2_ and *p_z_* orbitals of the metal; however, they support this idea only for strongly bent molecules, say for complexes showing *θ* ≤ 120°, a class our complexes do not belong to. According to the same authors our bromo-dimer may be classified as almost planar while the *β*-polymorph of the iodo-dimer belongs to the class with 150 ≤ *θ* ≤ 160°. 

**Figure 3:**
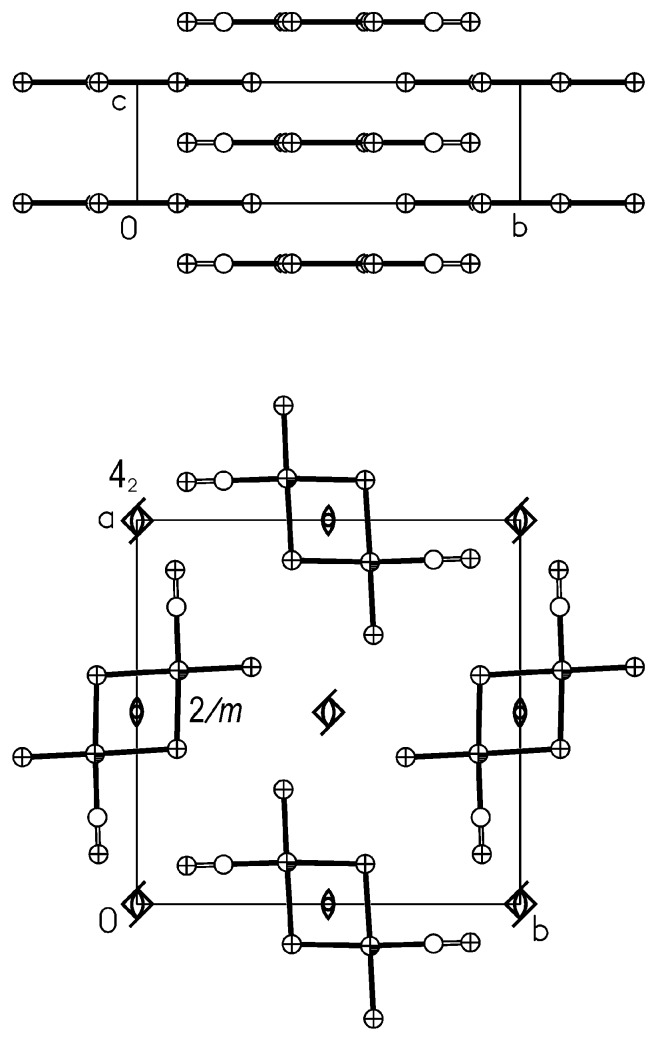
The crystal structure of *trans*-Pt_2_(*μ*-Cl)_2_Cl_2_(CO)_2_ viewed in the c (bottom) and in the **a** (top) directions. Some symmetry operators are reported.

As already discussed, with such a little bending the two metal centres do not get close enough to allow intra-molecular Pt···Pt bonding interaction. As intramolecular interactions do not appear to stabilize a particular form (planar or bent) with respect to the other, it is interesting to investigate if intermolecular contacts can play a role. The availability of the metric data for both geometries in the case of *trans*-Pt_2_(*μ*-I)_2_I_2_(CO)_2_ allows such a comparison. Let us start with a description of the crystal structures of the four compounds. The crystal structure of *trans*-Pt_2_(*μ*-Cl)_2_Cl_2_(CO)_2_ is shown in Figure 3. The molecules are stacked in columns running along c with a step corresponding to *c* (3.525 Å). Each column is hinged on a twofold axis crossing the inversion centres of the molecules, which this way, lie on a mirror plane (symmetry 2/*m*). Moreover, an equal number of inversion centres are placed on the stack axes in the middle of each couple of molecules, imposing perfectly eclipsed molecules. As shown at the bottom of Figure 3, each quartet of stacks is related by a screw 4_2_ so that the nearest stacks are staggered by a half step (top of the figure).

**Figure 4:**
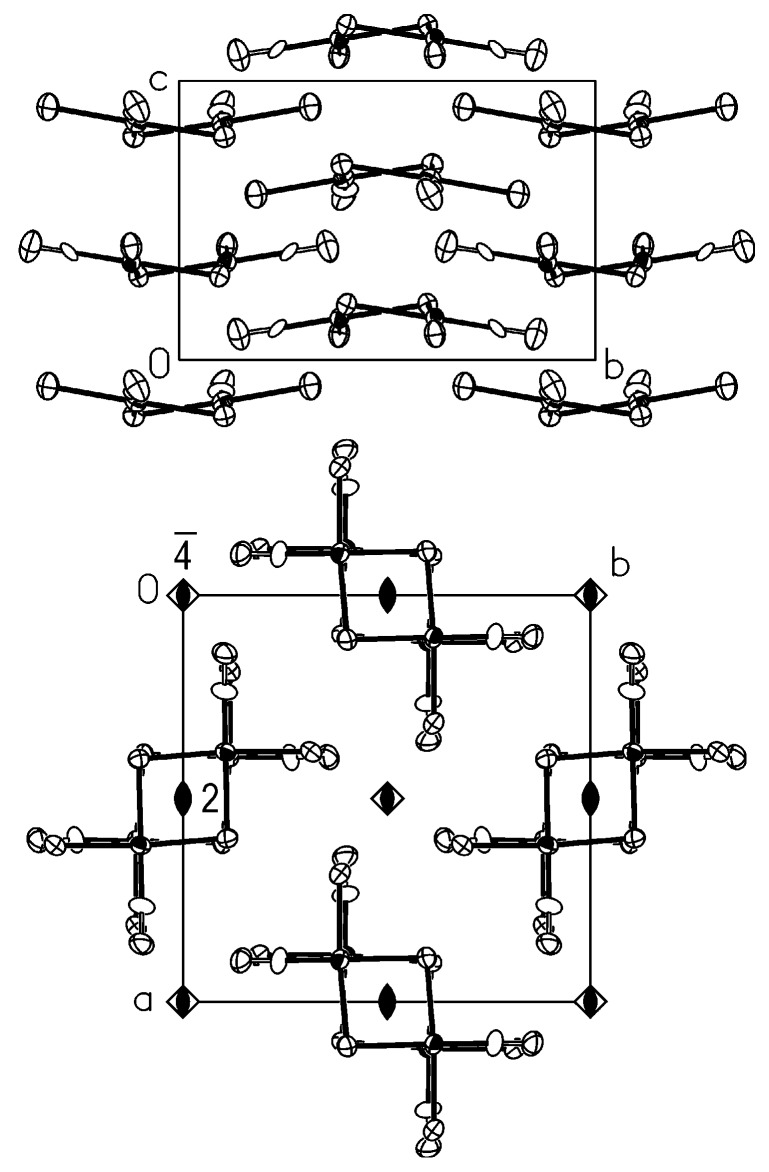
The crystal structure of *trans*-Pt_2_(*μ*-X)_2_X_2_(CO)_2_, X= Br, I(*β*) viewed in the **c** (bottom) and in the **a** (top) directions. Some symmetry operators are reported.

The crystal structure of the isotypic *trans*-Pt_2_(*μ*-Br)_2_Br_2_(CO)_2_ and *β*-*trans*-Pt_2_(*μ*-I)_2_I_2_(CO)_2_ is reported in Figure 4. Also in this case, the molecules are stacked in columns running along c with stacking steps 3.74 and 4.07 Å, respectively, and the columns are hinged on a simple twofold axis. This allows bent and not eclipsed molecules alternate in each column with CO groups pointing in perpendicular directions. The repetition occurs every two molecules and the vector c is approximately doubled (see the top of Figure 4). The quartets of columns are related by 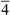
operations, which imply columns are staggered. The molecules of each column are bent in the same direction, while those of the nearest neighbour columns are bent in the opposite direction.

**Figure 5:**
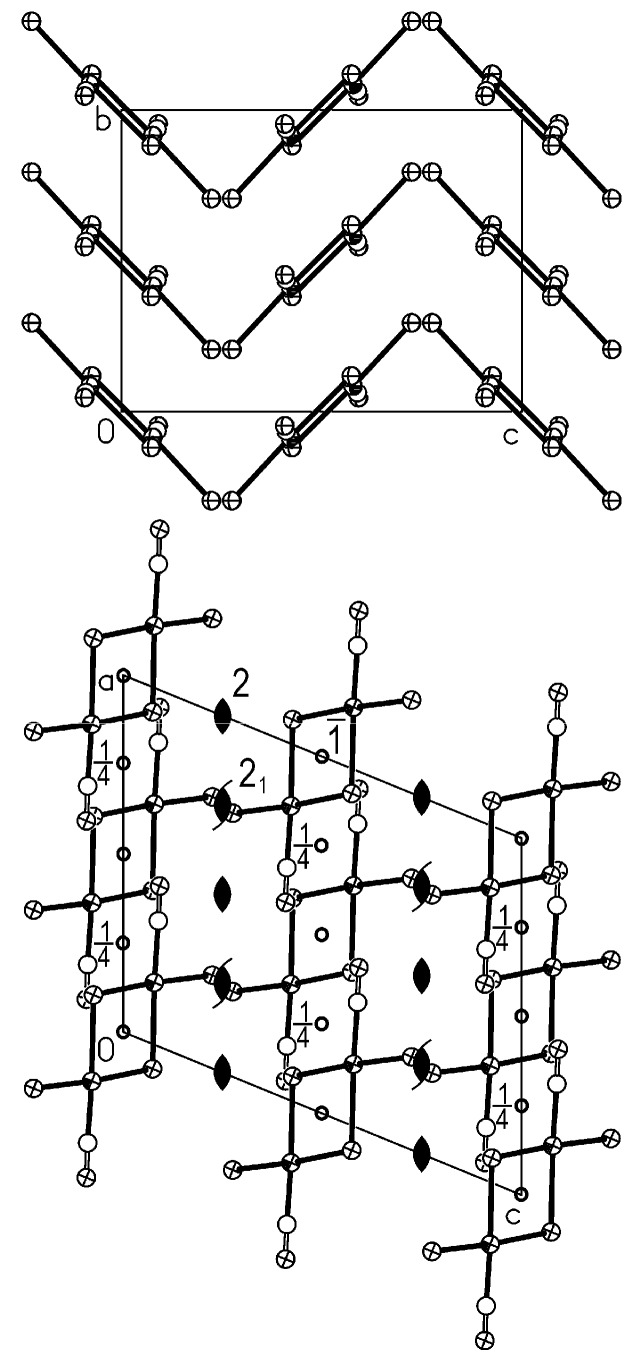
The crystal structure of *α*-*trans*-Pt_2_(*μ*-I)_2_I_2_(CO)_2_ viewed in the **b** (bottom) and in the a (top) directions. Some symmetry operators are reported.

Also the crystal structure of *α*-*trans*-Pt_2_(*μ*-I)_2_I_2_(CO)_2_, sketched in Figure 5, may be described in terms of stacks of molecules, but the planar molecules are heavily tilted, 47.6°, with respect to the column axis, running in this case along **b**. In the stacks only the inversion centres are present, as shown at the bottom of Figure 5. The nearest stacks in the **a** directions are related by the C centring of the lattice and so are staggered by *b*/2. On the other hand, the nearest rows of columns in the c direction, being related by twofold axes parallel to **b**, are staggered and show molecules tilted in the opposite direction. This feature produces the fishbone-like disposition of the stacks shown at the top of Figure 5.

As discussed so far, the crystal structures of the two complexes characterized in the course of this work show analogies with that of *trans*-Pt_2_(*μ*-Cl)_2_Cl_2_(CO)_2_, but are rather different from that of *α*-*trans*-Pt_2_(*μ*-I)_2_I_2_(CO)_2_.

The planar (*α*-) and bent (*β-*) forms of *trans*-Pt_2_(*μ*-I)_2_I_2_(CO)_2_ show a different molecular packing although in both structures the shortest intermolecular distances do not appear to imply significant interactions. In both polymorphs, having molecules organized in stacks, the intermolecular Pt···Pt distances, shorter in the *β*-modification, are however too long to support any hypothesis of significant metal-metal contacts. On the other hand, the slightly higher density (1.6 %) of the *α*-form is a signal of a more efficient system of intermolecular attractive interactions in this modification.

## 3. Experimental

### 3.1. General

All the operations were carried out using standard Schlenk tube techniques under dinitrogen or carbon monoxide. Solvents were purified according to standard methods. *cis*-PtBr_2_(CO)_2_ and *trans*-Pt_2_(*μ*-I)_2_I_2_(CO)_2_ were prepared according to the literature [16,18]. Platinum elemental analyses were carried out by calcining the products at 850 °C in a platinum crucible. Bromine elemental analyses were performed by the Volhard’s method previous reduction of the platinum to platinum(0). IR spectra were recorded with a FTIR Perkin-Elmer mod. Spectrum-One spectrophotometer.

### 3.2. Preparation of trans-Pt_2_(μ-Br)_2_Br_2_(CO)_2_

A pale yellow solution of *cis*-PtBr_2_(CO)_2_ (0.37 g, 0.9 mmol) in 1,2-dichloroethane (50 mL) was refluxed for about 7 h under a slight stream of N_2_. An orange solution was obtained whose IR spectrum showed an absorption at 2,127 cm^−1^ due to *trans*-Pt_2_(*μ*-Br)_2_Br_2_(CO)_2_. The solution was concentrated at reduced pressure till about half its volume. Addition of heptane (50 mL) caused the precipitation of an orange solid which was separated by filtration and dried *in vacuo* (144 mg, 42% yield). From the filtrate, cooled at about 0 °C, a second crop of the product was obtained (111 mg, 74% total yield). Elemental Analysis: Found (calc) %: Br: 41.4 (41.7); Pt: 51.0 (50.9). IR (PCTFE): 2,129 cm^−1^.

### 3.3. Crystallography

The X-ray diffraction measurements have been carried out at room temperature (*T* = 293 K) by means of a Bruker P4 diffractometer equipped with a graphite-monochromated Mo-*K_α_* radiation (*λ* = 0.71073 Å). The intensity data collection was carried out with the *w*/2*θ* scan mode, collecting a redundant set of data in order to check the diffraction symmetry and the reliability of the absorption correction procedure. Three standard reflections were measured every 97 measurements to check sample decay and equipment stability. The intensities were corrected for Lorentz and polarisation effects and for absorption by means of an integration method based on the crystal shape [19]. The structure solutions were obtained by means of the Patterson method and the refinements, based on full-matrix least-squares on *F*^2^, were done by means of SHELXTL programme [20].

Crystals of *trans*-Pt_2_(*μ*-Br)_2_Br_2_(CO)_2_ were grown by slow evaporation at room temperature of a solution of the product in CH_2_Cl_2_. A stick shaped sample 0.07 × 0.11 × 0.50 mm wide was sealed in a glass capillary. The cell parameters, calculated from the setting angles of 32 reflections having 5.4° < *θ* < 17.9°, are listed in Table 2, together with some other structural details. A total of 2148 intensities between 2.0° < *θ* < 30.0° was collected. The structure solution was obtained in the space group *P *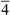 2_1_*c*. All the atoms were refined with anisotropic thermal parameters. The final refinement cycles gave the reliability factors listed in Table 2.

Crystals of *β*-*trans*-Pt_2_(*μ*-I)_2_I_2_(CO)_2_ separated out by cooling at −30 °C a solution containing Pt_2_I_4_(CO)_2_ and PtI_2_(CO)_2_ in toluene under CO. A stick shaped sample 0.03 × 0.05 × 0.30 mm wide was sealed in a glass capillary. The cell parameters, calculated from the setting angles of 47 reflections having 3.4° < *θ* < 17.1°, are listed in Table 2, together with some other structural details. A total of 1640 intensities between 2.3° < *θ* < 25.0° was collected. Since lattice parameters, intensity statistics and systematic extinctions suggested the structure was isotypic with the analogous bromide, the atomic parameters of that structure were used as a starting model for the refinement. The reliability factors obtained in the final refinement cycle are listed in Table 2.

Further details about collection and refinement with full lists of atomic parameters for both the crystal structures described in this paper have been deposited in the form of CIF files with the Cambridge Crystallographic Database. Dep. No. CCDC 830938 and 830939 for the bromide and the iodide, respectively. These data can be obtained free of charge via www.ccdc.cam.ac.uk/conts/retrieving.html (or from the CCDC, 12 Union Road, Cambridge CB2 1EZ UK; fax +44 1223 336033; Email: depositccdc.cam.ac.uk).

**Table 2:** Crystal data and structure refinements.

Compound	*trans*-Pt_2_(*μ*-Br)_2_Br_2_(CO)_2_	*β*-*trans*-Pt_2_(*μ*-I)_2_I_2_(CO)_2_
Empirical formula	C_2_Br_4_O_2_Pt_2_	C_2_I_4_O_2_Pt_2_
Formula weight	765.78	953.78
Crystal system	Tetragonal	Tetragonal
Space group	*P * 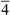 2_1_*c* (No. 114)	*P * 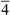 2_1_*c* (No. 114)
*a* / Å	11.522(1)	12.125(1)
*c* / Å	7.475(2)	8.132(1)
*U* / Å^3^	992.4(3)	1195.5(2)
*Z*	4	4
*D*calc / Mg·m^−3^	5.125	5.299
*μ* / mm^−1^	44.213	33.662
No. measured	2148	1640
No. unique [*R*_int_]	1033 [0.0513]	775 [0.1089]
No. parameters	48	48
*R*_1_, *wR*_2_ [*I*>2*σ*(*I*)]	0.0478, 0.0798	0.0517, 0.0857
*R*_1_, *wR*_2_ (all data)	0.0973, 0.0937	0.1325, 0.1093
Goodness of fit on *F*^2^	0.991	0.899

## 4. Conclusions

At the best of our knowledge, among the neutral [L_2_Pt(*μ*-X)_2_PtL_2_] complexes, *α*-*trans*-Pt_2_(*μ*-I)_2_I_2_(CO)_2_ [18] and *β*-*trans*-Pt_2_(*μ*-I)_2_I_2_(CO)_2_ (described in this work) represent the first case where planar and bent structural isomers are both structurally characterized. It is reasonable to suppose that the two polymorphs do not differ much in energy and that they are in rapid equilibrium in solution. Most likely, slight differences in the crystallization conditions (temperature, the presence of other species in solution) can favour a form with respect to the other. Although the intra-molecular Pt⋯Pt distance in the bent complex is shorter than in the planar one, it is however long enough to consider the presence of a metal-metal bond unlikely.

Only one comparable structural characterization of both bent and planar isomers has been reported for platinum(II) halo-bridged dimers: it concerns the cationic complex [H_2_bpyrPt(*μ*-Cl)_2_PtbpyrH_2_]^6+^ (bpyr = bis-pyrimidine) in the salts [Pt_2_Cl_2_bpyr_2_H_4_] [SbF_6_]_4_[Sb_2_F_11_]_2_·2HF (*θ* = 148.4°) and [Pt_2_Cl_2_bpyr_2_H_4_][Sb_2_F_11_]_6_·4HF (*θ* = 180°) [10]. However, unlike the neutral *α-* and *β-trans*-Pt_2_(*μ*-I)_2_I_2_(CO)_2_ species, the difference of the anionic counterpart in these two salts could justify a different stability of two cation forms.
